# Supplementary material for: Origin of agricultural plant pathogens: Diversity and pathogenicity of Rhizoctonia fungi associated with native prairie grasses in the Sandhills of Nebraska
Source: PLoS One. 2021 Apr 27;16(4):e0249335. doi: 10.1371/journal.pone.0249335 (PMC8078757; doi:10.1371/journal.pone.0249335)
Supplement: Attachment — Submitted filename: CoverLetter_PLoS_Response-to-Reviews_9-10-2020.pdf [file pone.0249335.s001.pdf]

September 10, 2020

To Samantha,

The following items were addressed in documents originally resubmitted on August 24, 2020. In this letter, we are providing additional documentation to state how these points were addressed in order to better clarify actions taken in response to the recommendations.

In response to the item "2.1. Please provide an amended Funding Statement declaring this commercial affiliation", we provided information in the online submission form explaining that the co-author with a commercial affiliation was not affiliated with that company during the time that he completed the work related to this manuscript and that the commercial company is simply his current address. Thus, the company, Terramera Inc., had no role in the study design, data collection and analysis, decision to publish, or preparation of the manuscript and provided no financial support in any form. Similarly for item "2.2. Please also provide an updated Competing Interests Statement", no change seems needed since there is no commercial affiliation with the research. Please provide further clarification about the changes you would like us to make and where those changes should be made if you believe this response is insufficient.

In response to items "2.1. You may seek permission from the original copyright holder of Figure 1 to publish the content specifically under the CC BY 4.0 license" and "2.2. If you are unable to obtain permission from the original copyright holder to publish these figures under the CC BY 4.0 license or if the copyright holder's requirements are incompatible with the CC BY 4.0 license, please either i) remove the figure or ii) supply a replacement figure that complies with the CC BY 4.0 license", we would like to draw your attention to the new Figure 1 that was uploaded on August 24, 2020 as a replacement for the previous figure. This change was noted within the online form where asked about "Copyrighted Figures" and our response was "No, no figures use a copyrighted image. The county boundary file we used to create the map in Figure 1 was obtained from nebraskamap.gov, which specifically states that the county boundary file has no copyright.". If you feel this response is insufficient, please provide greater detail in the explanation about what change is needed in order to show that this change was made.

Sincerely,

Sydney E. Everhart
Associate Professor
Department of Plant Pathology
University of Nebraska
Lincoln, NE 68583-0722
Phone: (402) 472-2879
everhart@unl.edu
Website: everhart.unl.edu
