# Supplementary material for: Origin of agricultural plant pathogens: Diversity and pathogenicity of Rhizoctonia fungi associated with native prairie grasses in the Sandhills of Nebraska
Source: PLoS One. 2021 Apr 27;16(4):e0249335. doi: 10.1371/journal.pone.0249335 (PMC8078757; doi:10.1371/journal.pone.0249335)
Supplement: Attachment — Submitted filename: Comments from reviewer 03122021.docx [file pone.0249335.s002.docx]

Response to comments from Reviewer 4:

- Line 131-134 - kindly re-frame this sentence

Reframed the sentence

- 178 - Kindly mention the percentage of alcohol

Done

- Kindly abbreviate the genus name after first use in text

Done

- Line 311-312 kindly re-write this sentence

Done

- Line 339 and 341 - Kindly check the spelling of Conetainers

Although it is possible to find the word “conetainers” used in manuscripts, most web searches show it as a hyphenated word. We have updated our manuscript to use the hyphenated version.
